# The Legend of ATP: From Origin of Life to Precision Medicine

**DOI:** 10.3390/metabo12050461

**Published:** 2022-05-20

**Authors:** Xin-Yi Chu, Yuan-Yuan Xu, Xin-Yu Tong, Gang Wang, Hong-Yu Zhang

**Affiliations:** 1 Hubei Key Laboratory of Agricultural Bioinformatics, College of Informatics, Huazhong Agricultural University, Wuhan 430070, China; chuxiyi@nbu.edu.cn (X.-Y.C.); xuyuan2@webmail.hzau.edu.cn (Y.-Y.X.); tongxinyu@webmail.hzau.edu.cn (X.-Y.T.); wanggang2020@webmail.hzau.edu.cn (G.W.); 2 Qian Xuesen Collaborative Research Center of Astrochemistry and Space Life Sciences, Institute of Drug Discovery Technology, Ningbo University, Ningbo 315211, China

**Keywords:** adenosine triphosphate (ATP), origin of life, precision medicine, drug discovery, phase separation, hydrotrope

## Abstract

Adenosine triphosphate (ATP) may be the most important biological small molecule. Since it was discovered in 1929, ATP has been regarded as life’s energy reservoir. However, this compound means more to life. Its legend starts at the dawn of life and lasts to this day. ATP must be the basic component of ancient ribozymes and may facilitate the origin of structured proteins. In the existing organisms, ATP continues to construct ribonucleic acid (RNA) and work as a protein cofactor. ATP also functions as a biological hydrotrope, which may keep macromolecules soluble in the primitive environment and can regulate phase separation in modern cells. These functions are involved in the pathogenesis of aging-related diseases and breast cancer, providing clues to discovering anti-aging agents and precision medicine tactics for breast cancer.

## 1. Introduction

The origin of life is one of the most significant and fundamental issues in science. In this controversial area, numerous “starting points” of life have been proposed. Different from the biological macromolecules that are conventionally concerned, Sharov focuses on small molecules with catalytic functions and proposed a coenzyme world model: the coenzyme-like molecules (CLMs) attached to the oil droplets constitute the earliest system capable of primitive metabolism and evolvable self-reproduction [1,2]. Although such a system has not been proven, coenzymes’ structural simplicity and functional importance make CLMs an attractive starting point for life.

Among the various coenzymes, adenosine triphosphate (ATP) is an especially attractive one. ATP is one of the most abundant components in the modern cell, with a concentration of up to 10 mmol/L [3]. Coinciding with its high concentration, ATP is indeed versatile. As a coenzyme, ATP is bound by hundreds of protein structures and, thus, is involved in many metabolic pathways. Since first isolated from muscle and liver extracts by Karl Lohmann in 1929, ATP was regarded as life’s energy reservoir [4]. ATP is a component of RNA and a substrate for the first step of protein synthesis. Through protein phosphorylation reaction, ATP participates in the signaling of key bioprocesses. ATP can also work as a transmitter in intercellular purinergic signaling [5]. Recently, ATP was found to have a role as a hydrotrope and regulate cellular compartmentalization [6]. 

From the beginning to the current scene, ATP plays multiple critical roles in the drama of life. In this paper, we will show why ATP is so important to the origin of life and how the critical functions of ATP open up a new area of biomedicine.

## 2. Prebiotic Synthesis of ATP

ATP may exist on the primitive Earth. ATP is composed of adenine, a ribose, and a triphosphate group. The prebiotic synthesis of adenine and ribose has been extensively studied and excellently reviewed in a recent work by Yadav et al. [7]. The soluble phosphorus required for the synthesis of triphosphate groups may be provided by the phosphite contained in the extraterrestrial schreibersite or the polyphosphates produced by volcanic activity [8,9], which can be converted to orthophosphate in plausible prebiotic environments [8]. One obstacle to the synthesis of nucleotides is that the condensations of the three components are thermodynamically unfavorable in water [10]. A possible solution to this challenge is adsorbing the reactants on mineral surfaces, and then carrying out the condensations during a drying process. Using such a strategy, Akouche et al. synthesized adenosine monophosphate (AMP) from adenosine, ribose, and potassium dihydrogenophosphate on the amorphous silica at 70 °C [11]. AMP can be further phosphorylated into ADP and ATP in the presence of hydroxyapatite and cyanate at room temperature [12], or by reacting with metaphosphoric acid under the catalysis of metal ions [13,14]. Nickel was found to be the most efficient metal catalyst for ATP synthesis, which may have been brought to the Hadean Earth by meteorites [15]. These reactions do not rely on harsh physical and chemical conditions and thus may be feasible on the primitive Earth.

## 3. ATP as the Cofactor of Primitive Proteins

Being biological energy currency is the most well-known biological function of ATP, but may not be the first [16,17]. However, even without this function, ATP is still vital to life. Although RNA world theory has been debated since it was proposed in the 1960s [18], it is still one of the most widely accepted hypotheses about the origin of life [19]. This theory proposed a life form in which RNA takes the responsibility of carrying genetic information and catalyzing biochemical reactions. As a basic monomer for RNA polymerization, ATP lays the foundation of the RNA world. Forty-five years ago, White suggested nucleotide-containing cofactors, such as ATP, nicotinamide adenine dinucleotide (NAD), flavin adenine dinucleotide (FAD), and coenzyme A, were firstly the cofactors of the RNA enzymes; when the protein enzymes replace the RNA ones, these nucleotide-containing cofactors still kept their position [20]. White’ s opinion, as we will discuss below, has a significant inspiration for studies about protein origin and evolution.

In the field of protein origin and evolution, it is generally considered that the sequences and architectures that originated earlier are more widely shared by extant proteins [21,22]. Following this principle, we have pinpointed the early cofactor–protein interactions through analyzing the distribution patterns of cofactors in the protein structure space [23,24]. The cofactor-structure mapping shows a power–law relationship: most cofactors bind only one structure, while a few cofactors can bind tens of structures. ATP was found to be the most prevalent cofactor in protein structure space, and, thus, should be one of the earliest cofactors used by proteins. Furthermore, we inferred that the most ancient protein using ATP belongs to P-loop containing nucleoside triphosphate hydrolases (P-loop NTPases, pertaining to c.37 protein fold, defined by the Structural Classification of Proteins (SCOP) database [25]). Other nucleotide-containing cofactors, such as NAD and FAD, were also found in many protein structures, showing their early co-operation with proteins [23]. The corresponding earliest protein architectures belong to NAD-binding Rossmann-fold domains (c.2 fold) and FAD/NAD-binding domain (c.3 fold), respectively [23].

This result is consistent with the previous sequence- and structure-based protein origin studies. Sobolevsky and Trifonov identified short protein sequence fragments that are conserved among the 131 prokaryotic genomes available at the time [21]. Most of these fragments belong to the Walker A motif [26], which is the ATP binding region of P-loop NTPases. Based on different protein structure classification schemes, Caetano-Anollés’ group built phylogenomic trees for protein structures [22,27,28,29]. P-loop NTPases proteins are located at the roots of these trees, indicating the ancient origin of this structure. Combining the sequence and structure information, Alva et al. identified 40 protein fragments that were inferred to be the remnants of primitive proteins, and one of them is the component of P-loop NTPases [30].

## 4. Why ATP? ATP Facilitated the Origin of Protein

The above findings suggest that primitive proteins bind ATP and keep this feature to this day. A question naturally arises: why was ATP used by the most ancient proteins? A possible answer resides in the fact that ATP promotes protein folding and folded proteins are more resistant to degradation and more likely to be functional than the unfolded ones. The free energy released during ATP–protein binding is 10~15 kcal/mol, which is close to the free energy of protein folding (10~20 kcal/mol) [31,32]. This energy may help the folding of the primitive proteins [23]. Therefore, it was proposed that the most ancient protein structures may be selected from random peptide sequences by cofactors, such as ATP [23]. Tokuriki and Tawfik presented a similar opinion about protein origin, which suggested that ligand binding selected the primitive protein structures and functions [33]. In support of this idea, ATP was indeed found to facilitate the folding of E. coli glyceraldehyde-3-phosphate dehydrogenase [34].

The above hypotheses about protein origin are in accordance with experimental observations. To investigate the occurrence frequency of functional/folded proteins in random sequences, Keefe and Szostak performed an in vitro selection using ATP as the bait. As a result, four families of ATP-binding proteins were selected from 6 × 10^12^ random sequences [35]. One of the proteins was crystallized and its structure is closest to c.37 fold [28], which is just the most ancient protein architecture inferred by ATP distribution patterns in the protein structure space [23]. Our group carried out in vitro selection for ATP-binding protein with a different random protein library which is composed of only 15 kinds of amino acids [36]. The excluded five amino acids, i.e., Phe, Cys, Met, Tyr, and Trp, were considered to be not abundant in the primitive Earth and, thus, may not exist in the early proteins [37]. The ATP-binding protein we obtained was found to have NTPases activity, which implied another role that ATP played in protein origin and evolution. A major part of modern proteins are enzymes; some of them catalyze energetically uphill reactions. For these reactions, external energy sources are also necessary. The high-energy phosphate bond contained in ATP can meet this demand. Enzymes with ATPase activity enable organisms to use ATP as an energy source to drive more metabolic reactions, laying the metabolic foundation for more complex and delicate life forms.

## 5. Why ATP? ATP as a Hydrotrope

Other cofactors, e.g., NAD, may also facilitate protein folding [38] or provide energy for biochemical reactions (in the reduced form, NADH) [39]. So, why is ATP the one that binds the most prevalent protein structures and is likely the earliest cofactor used by proteins? Recently, a new property of ATP was revealed: high-level ATP can function as a hydrotrope to prevent protein aggregation [6]. Most proteins only maintain activity in the solubilized state; over-aggregation can cause protein precipitation. We argue that this function is especially significant for the origin of proteins. There is an opinion that many primitive proteins are intrinsically disordered [40], and these proteins are more prone to aggregate or even precipitate than globular ones [41]. Thus, the ATP-like solubilization-facilitating property is crucial to protein origin. RNA-binding protein fused in sarcoma (FUS) is a model intrinsically disordered protein. At the millimolar level, ATP can prevent the aggregation of FUS and even dissolve FUS amyloid fibers in higher concentrations. A recent molecular dynamics study revealed the mechanism underlying these processes: the hydrophobic adenine head of ATP pretended to contact the core of FUS aggregates, while its hydrophilic phosphoric acid tail was exposed to the external solvent, which promoted the dissolution of the aggregates [42]. This property has not been observed for other cofactors, manifesting the irreplaceable role of ATP in the origin of life.

In modern cells, the hydrotrope function of ATP is still significant. To coordinate the huge amount of contents inside the crowded intracellular space, functionally related proteins can condense through liquid–liquid phase separation (LLPS) [43]. In cytoplasm, the formed LLPS droplets, also called membrane-less organelles, help to maintain the efficiency of molecular machines [44]. However, LLPS is thermodynamically imbalanced. To minimize the system’s free energy, the separated phases tend to further condense. So, without external regulation, the separated phases do not disappear spontaneously, which may generate harmful amyloids [45]. ATP has been proven to prevent the formation of protein LLPS droplets and dissolve previously formed ones [6], and is considered to be a key regulator of cellular LLPS [43,46]. 

LLPS also exists inside the nucleus and is regulated by ATP. During gene transcription and genome replication, numerous biomolecules agglomerate and form liquid–liquid separated phases, which remodeled the structure of the chromosome to ensure proteins access the genome and perform their tasks in a relatively stable environment [47]. Hormones, such as estrogen, can induce chromatin remodeling and specific gene transcription accompanying intranuclear phase separation. Wright et al. observed an ATP level increase in the nucleus of estrogen-stimulated breast cancer cells and further showed that this intranuclear ATP is required for chromatin remodeling and gene transcription [48]. In a follow-up paper, Wright et al. proposed that, as a hydrotrope, ATP can regulate the production, maintenance, and dissolution of dynamic phase separation in the nucleus [49]. They also suggested that ATP may influence the concentration of Mg^2+^, and the latter is well known to influence the solubility of chromatin.

As shown in the above descriptions, ATP acts as a hydrotrope to participate in fundamental cellular physiological processes through regulating biomolecular condensates. These advances not only explain why ATP remains at high concentrations in cells, but also bring inspirations to biomedicine, especially in combating aging-related diseases and breast cancer.

## 6. Implications of ATP in Aging-Related Diseases

Mitochondria, the main source of cellular ATP, has been considered to be related to aging for decades [50]. A common view is that the mitochondrial genome accumulates harmful mutations with age, which damages mitochondrial function, leads to cell energy shortage, and further causes aging pathologies. Consistent with this point of view, a decline in ATP with age has been observed in different animal and human investigations [51] and is related to aging pathologies, such as sarcopenia [52], heart failure [53], and neurodegenerative diseases [54,55,56].

The hydrotrope character of ATP is also involved in aging. As mentioned above, ATP is a key regulator of biomolecules. Dysregulation of protein condensates causes harmful protein aggregation, misfolding, and dissolution [44], and the further induced loss of proteostasis is a hallmark of aging [57]. A typical example is the generation of Aβ amyloid deposition, which plays an important role in the pathogenesis of neurodegenerative diseases. ATP can prevent and even dissolve different types of Aβ amyloid aggregation [6,58,59], and may reduce the wrong folding of Aβ [60]. A recent molecular dynamics simulation study found that ATP has the potential to inhibit the aggregation of human islet amyloid polypeptide, which can lead to type II diabetes mellitus [61].

The association between ATP and the aging-related diseases naturally sparked the idea of treating these diseases by maintaining the normal concentration of ATP. In the discovery of neurodegenerative disease treatment, a “brain energy rescue” strategy has been practiced for several years [62]. A series of compounds capable of elevating ATP cellular, animal, and human experiments have been reported [63,64,65]. A more detailed introduction of these studies could be found in our previous paper [66].

Moreover, the decrease in cellular NAD, which is another nucleotide-containing cofactor, has been found to be deeply involved in multiple aging-related cellular processes, and restoring NAD level has emerged as a helpful therapy for aging-related diseases [67]. Reduced NAD (NADH) is a central hydride donor in mitochondrial ATP synthesis. A study observed that ATP level decreased following the NAD decline in toxic prion-protein-treated neurons [68], suggesting that ATP is involved in the NAD-related aging mechanism. Thus, we consider that ATP may be partially responsible for NAD-augmentation-based aging prevention.

## 7. Implications of ATP in Precision Medicine of Breast Cancer

Estrogen is the most important hormone that directly stimulates the growth and development of the breast, and it also plays a vital role in the occurrence and development of estrogen-receptor-positive breast cancer. As mentioned above, ATP can regulate the estrogen-induced production, maintenance, and dissolution of dynamic phase separation in the nucleus [48]. These processes are necessary for the expression of estrogen-regulated genes and important in the proliferation of breast cancer cells. Moreover, Wright et al. found that the concentration of ATP in the nucleus significantly increased 30 min after the hormone stimulation, while the level of ATP in the mitochondria or cytoplasm did not change, suggesting a source of ATP inside the nucleus [48]. Nudix hydrolase 5 (NUDIX5, also called NUDT5) has been identified as a key factor in the production of ATP in the nucleus, which can use intranuclear ADP-ribose (ADPR) as the substrate to synthesize ATP in the presence of diphosphate [48]. Further studies on NUDT5 showed that both the intranuclear ATP level increase and the estrogen-regulated gene transcription depended on the activity of NUDT5. These findings revealed that ATP functions as an estrogen coactivator by mediating phase separation in the nucleus [49,69].

This ATP new function brings a novel strategy for estrogen-related disease treatment, that is, suppressing the activity of estrogen by decreasing the concentration of ATP, which may be achieved by inhibiting NUDT5. Page et al. discovered a series of NUDT5 inhibitors and proved that these compounds can repress the nuclear ATP production, and further restrain hormone signal transduction and cell proliferation in breast cancer cells [70]. However, these inhibitors have not yet entered clinical trials, which means that they cannot be clinically used in the near future. To accelerate the development of drugs based on this new concept, we resorted to a drug-repositioning strategy. Through bioinformatic analysis, molecular simulation, and cell-level experiments, seven approved drugs were identified as potential NUDT5 inhibitors and were proved to have a cytotoxic effect on estrogen-receptor-positive breast cancer cell line MCF7 [71]. These drugs are of apparent interest for further evaluation.

In recent years, precision medicine has attracted much attention in the area of cancer therapy. Biomarkers are key components for precision medicine, which help to classify patients according to different expectations for prognosis, treatment response, and disease susceptibility [72]. Prognostic biomarkers can provide information about overall cancer outcomes to facilitate cancer diagnosis [73]. For estrogen-receptor-positive breast cancer, there remains a lack of commonly accepted prognostic biomarkers. Our group found that estrogen-receptor-positive breast cancer patients with low-level NUDT5 expression had significantly longer survival times compared with high-NUDT5-level counterparts [74]. In comparison, this phenomenon cannot be observed in estrogen-receptor-negative breast cancer patients. Together, all these results exhibited the potential of NUDT5 as a drug target and a prognostic biomarker in precision medicine of breast cancer.

## 8. Summary and Outlook

ATP takes a significant part in the origin of life. ATP constituted the ancient ribozymes and facilitated the generation of the earliest proteins. The energy contained in its phosphate bond may drive the prebiotic metabolic reactions. The amphiphilic structure makes the molecule an effective hydrotrope, which helps to maintain the solubility of primitive biomolecular condensates. The legend of ATP continues to this day. In modern organisms, ATP helps to delay the inevitable destiny of life—aging, and may contribute to precision medicine by inspiring the discovery of therapeutic drugs and prognostic biomarkers for breast cancer.

The primary progress introduced in the current paper indicates the potential of ATP in precision medicine. However, from now, the understanding of ATP as a hydrotrope is still insufficient. Most of the knowledge on this function was obtained from in vitro and cellular studies. The local environment regulation in live cells is more complicated. Take the estrogen-receptor-regulated transcription as an example, multiple regulators are recruited and arranged by scaffold molecules, such as SRA RNA, to form functional transcription complexes [75]. This process is a key part of the pathogenesis of breast cancer. Understanding how ATP is involved in such processes could be a challenging topic. Whether the desired effect can be achieved in living organisms needs much more research effort.

Moreover, most functions of ATP are very fundamental, which means activating or inhibiting related targets may cause prevalent perturbation. During the protein synthesis, ATP not only is utilized as the energy reservoir and substrate of tRNA aminoacylation, but also as a regulator. In bacteria, the activity of ribosome RNA promoters is correlated with the concentration of initiating NTP and, on most occasions, of ATP [76]. In this way, ribosome biogenesis and protein synthesis are physiologically connected with cellular energy status. However, an excess of ATP will arrest too much Mg^2+^, and inhibits ribosome biogenesis and cell growth, because Mg^2+^ is necessary to maintain ribosome structure [77]. How to accurately control the effect of ATP could be a challenge. Therefore, it seems that there is a long way to the finale of the legend of ATP.
